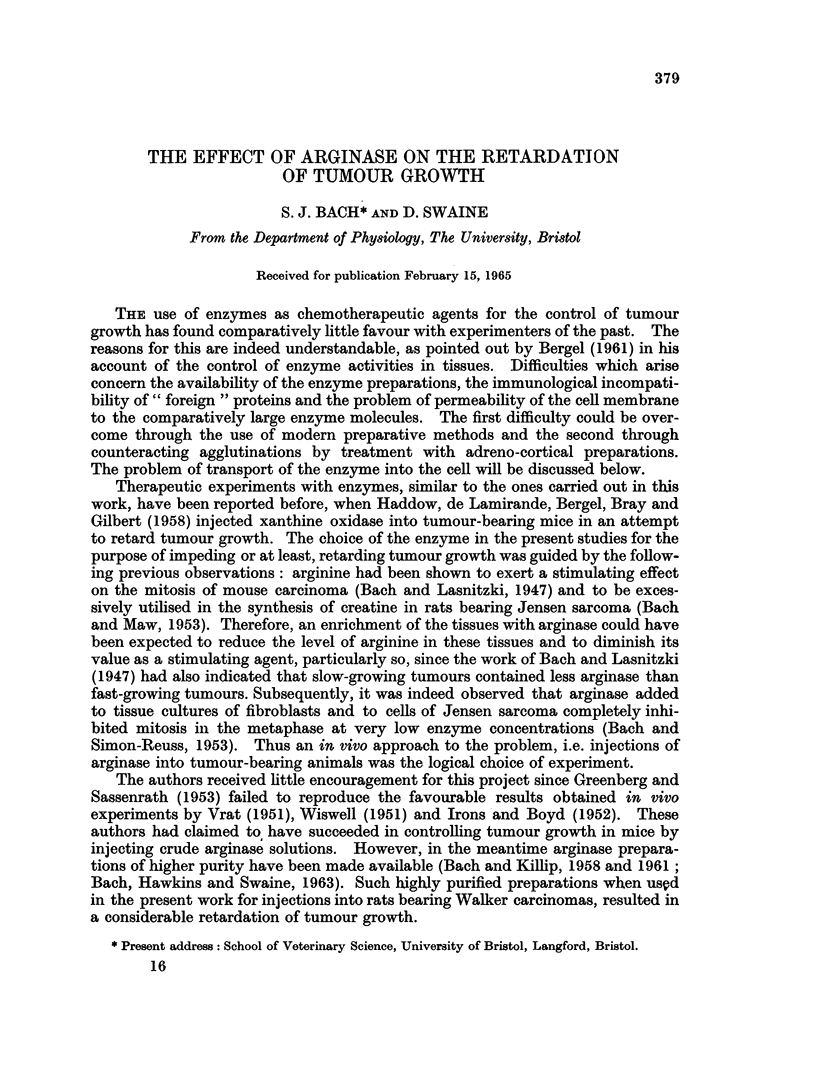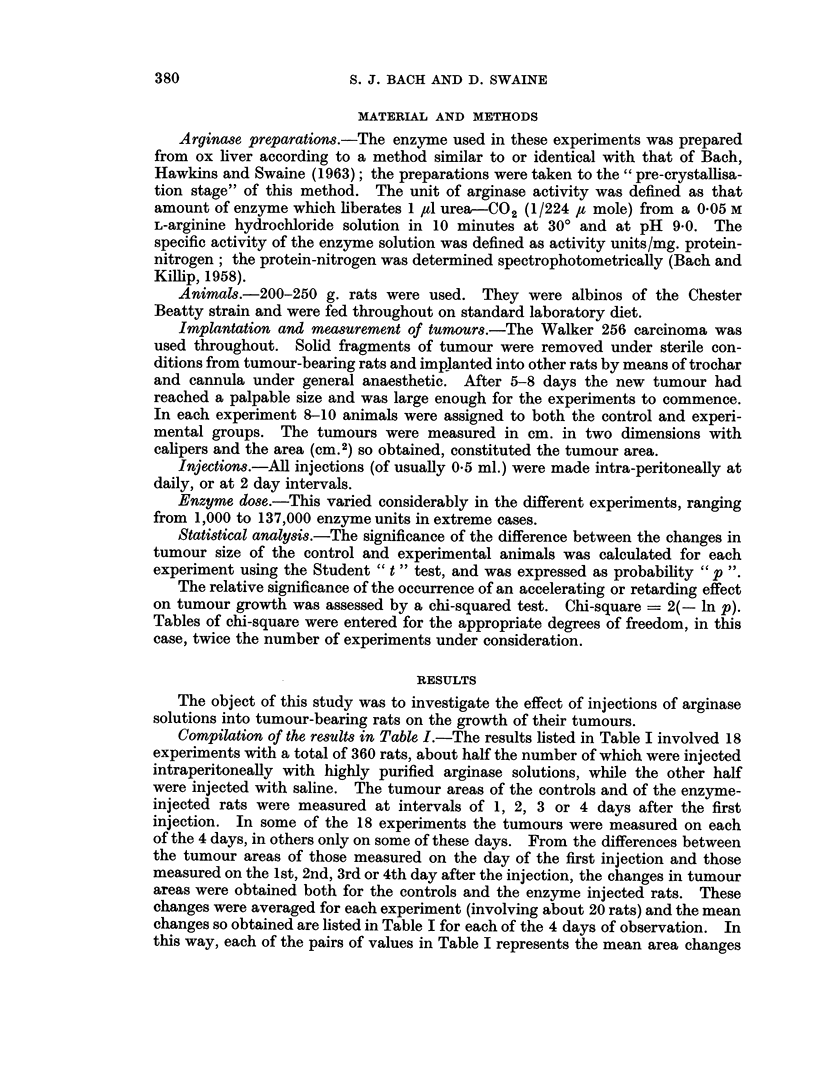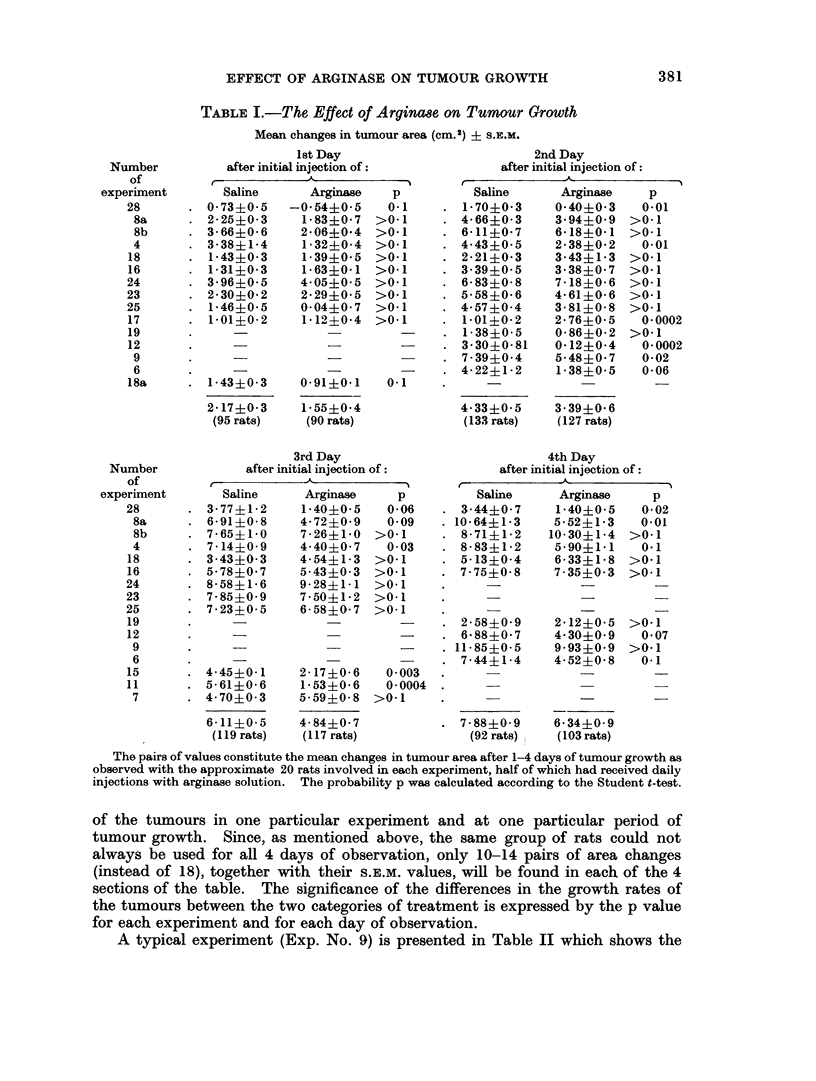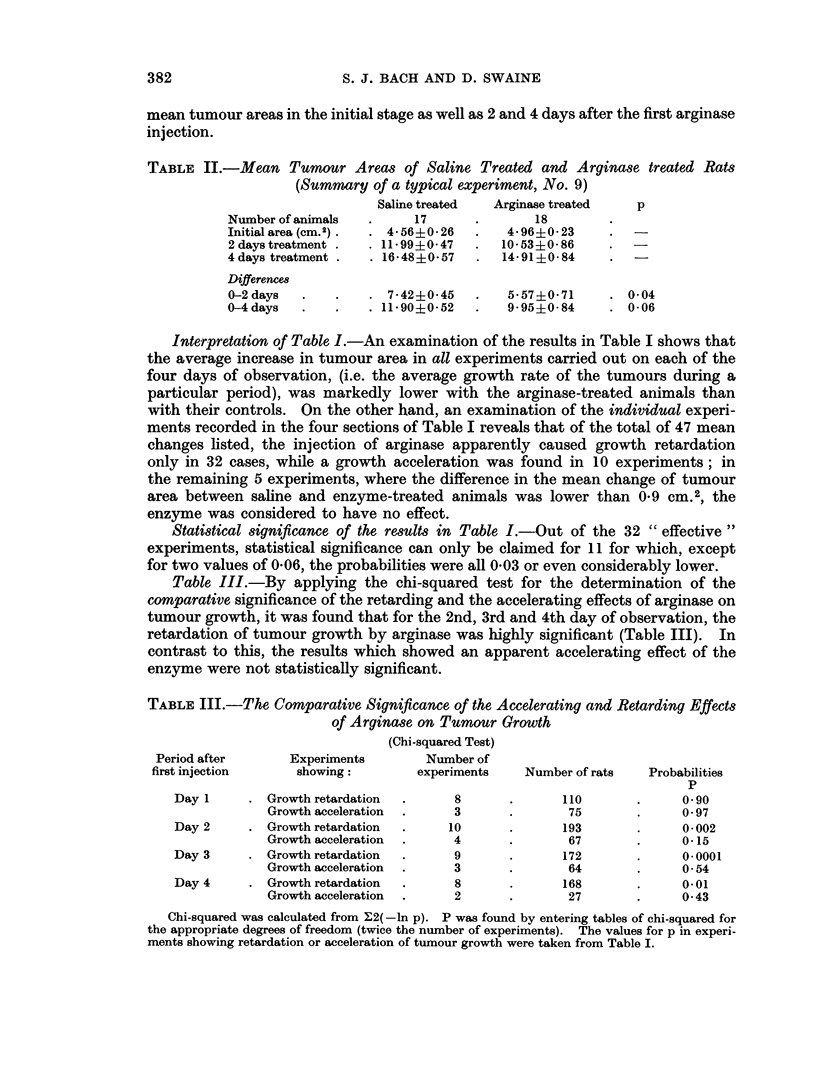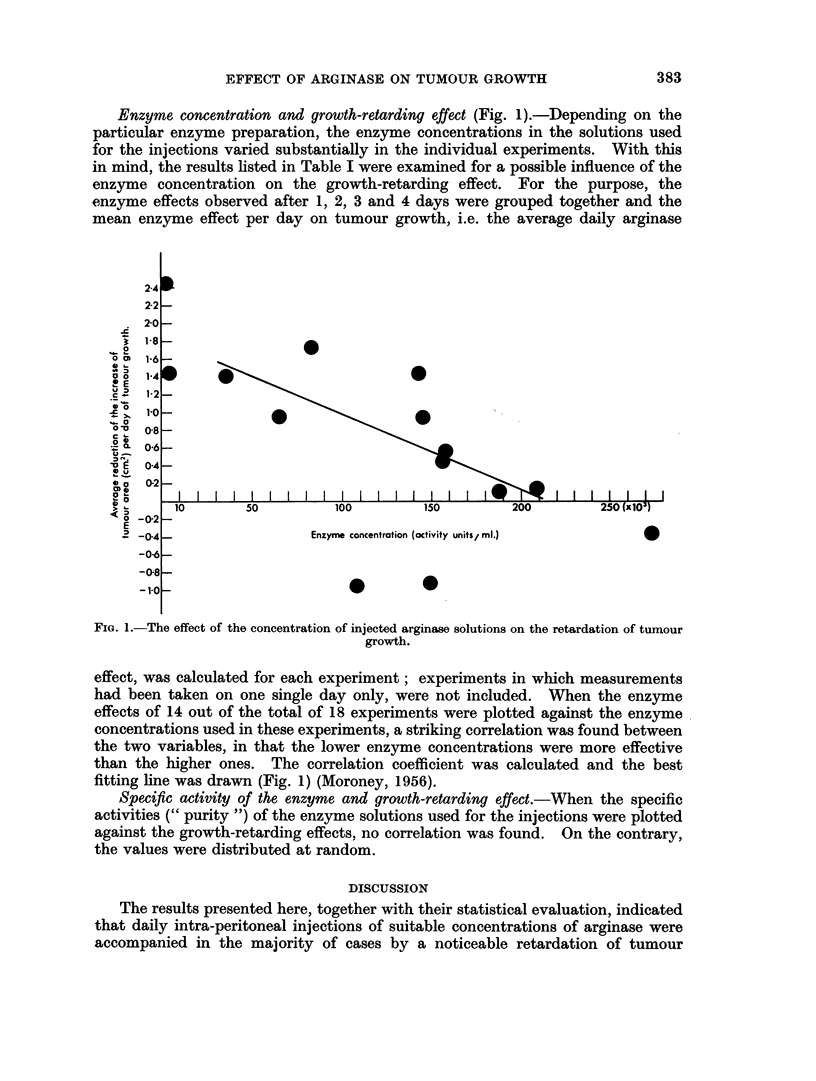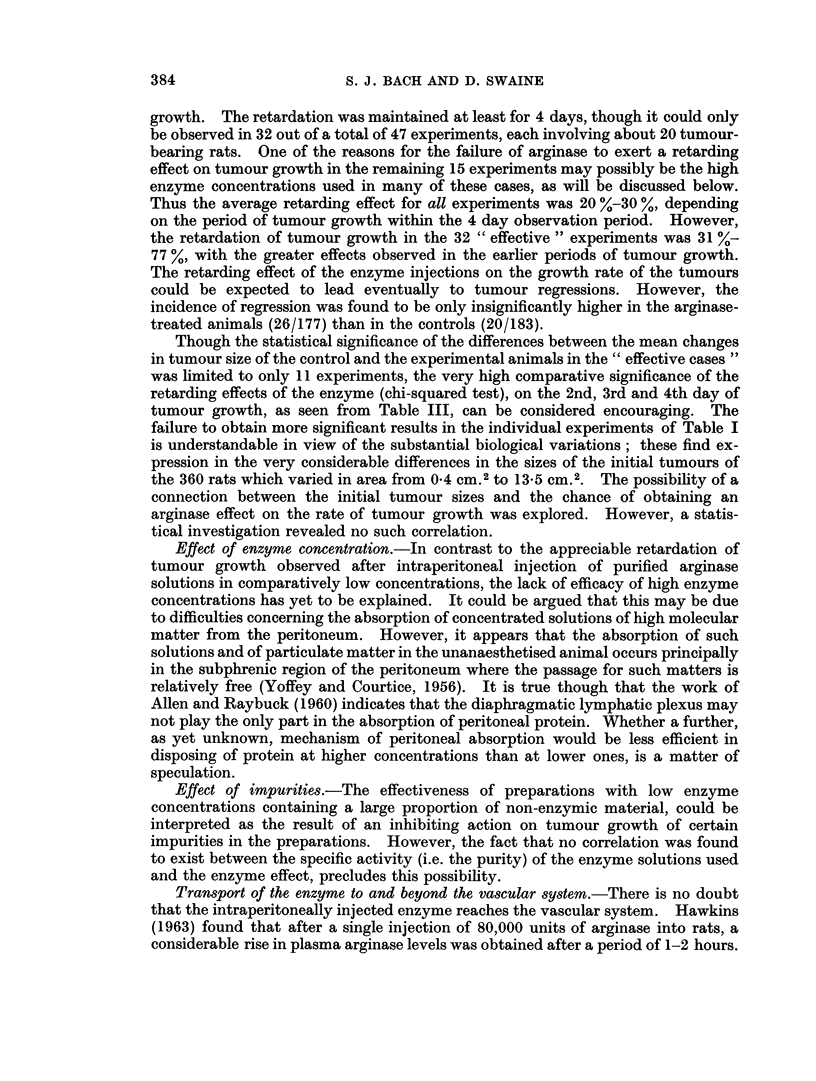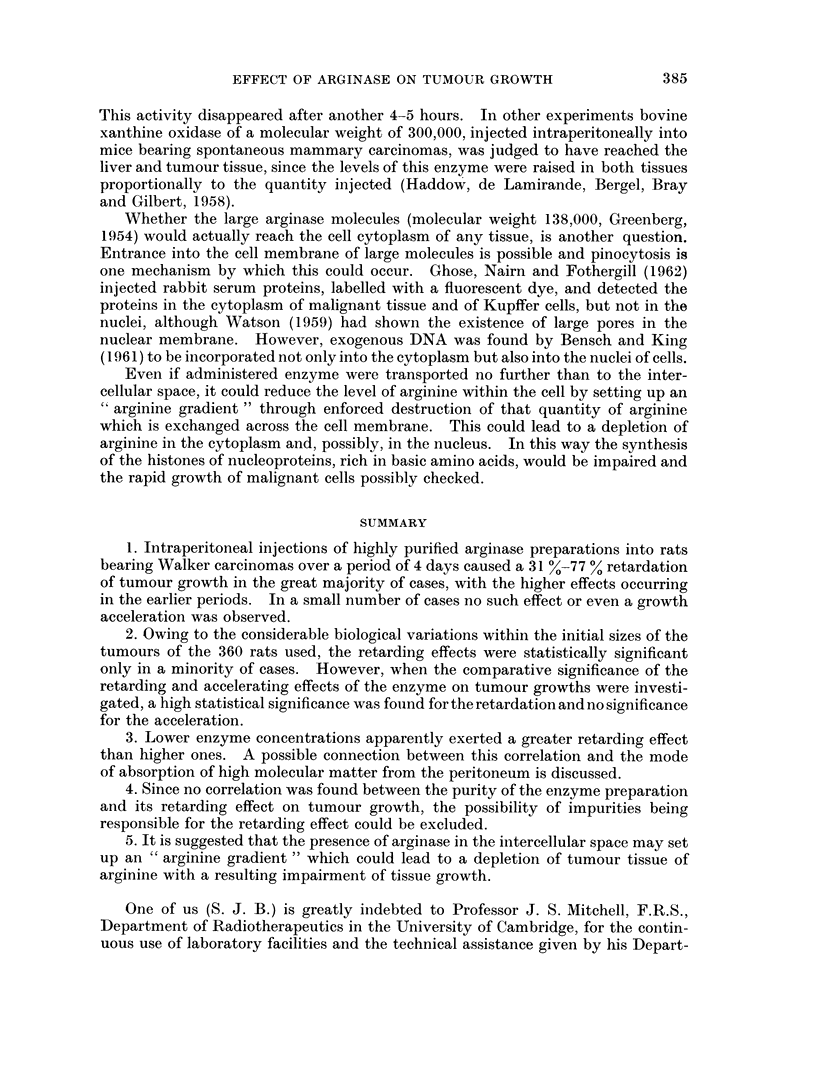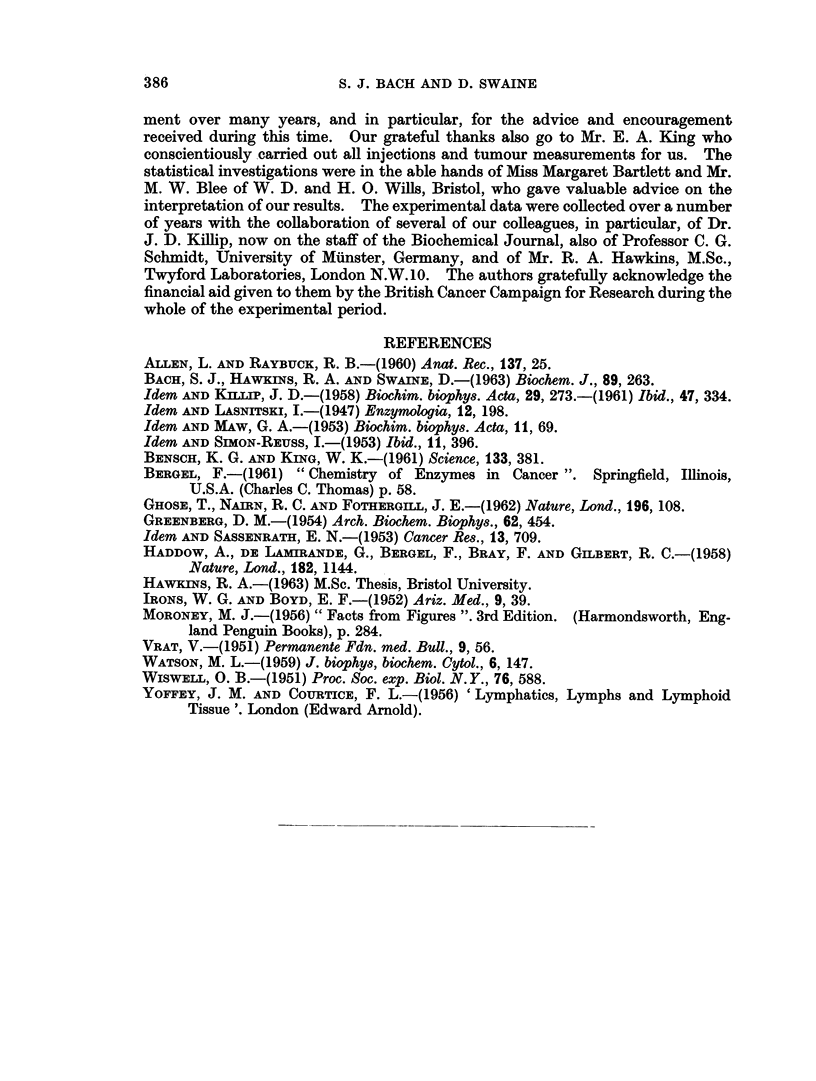# The Effect of Arginase on the Retardation of Tumour Growth

**DOI:** 10.1038/bjc.1965.45

**Published:** 1965-06

**Authors:** S. J. Bach, D. Swaine


### THE EFFECT OF ARGINASE ON THE RETARDATION OF TUMOUR GROWTH

S. J. BACH* AND D. SWAINE

*From the Department of Physiology, The University, Bristol*

Received for publication February 15, 1965

THE use of enzymes as chemotherapeutic agents for the control of tumour growth has found comparatively little favour with experimenters of the past. The reasons for this are indeed understandable, as pointed out by Bergel (1961) in his account of the control of enzyme activities in tissues. Difficulties which arise concern the availability of the enzyme preparations, the immunological incompatibility of " foreign " proteins and the problem of permeability of the cell membrane to the comparatively large enzyme molecules. The first difficulty could be overcome through the use of modern preparative methods and the second through counteracting agglutinations by treatment with adreno-cortical preparations. The problem of transport of the enzyme into the cell will be discussed below.

Therapeutic experiments with enzymes, similar to the ones carried out in this work, have been reported before, when Haddow, de Lamirande, Bergel, Bray and Gilbert (1958) injected xanthine oxidase into tumour-bearing mice in an attempt to retard tumour growth. The choice of the enzyme in the present studies for the purpose of impeding or at least, retarding tumour growth was guided by the following previous observations : arginine had been shown to exert a stimulating effect on the mitosis of mouse carcinoma (Bach and Lasnitzki, 1947) and to be excessively utilised in the synthesis of creatine in rats bearing Jensen sarcoma (Bach and Maw, 1953). Therefore, an enrichment of the tissues with arginase could have been expected to reduce the level of arginine in these tissues and to diminish its value as a stimulating agent, particularly so, since the work of Bach and Lasnitzki (1947) had also indicated that slow-growing tumours contained less arginase than fast-growing tumours. Subsequently, it was indeed observed that arginase added to tissue cultures of fibroblasts and to cells of Jensen sarcoma completely inhibited mitosis in the metaphase at very low enzyme concentrations (Bach and Simon-Reuss, 1953). Thus an *in vivo* approach to the problem, i.e. injections of arginase into tumour-bearing animals was the logical choice of experiment.

The authors received little encouragement for this project since Greenberg and Sassenrath (1953) failed to reproduce the favourable results obtained *in vivo* experiments by Vrat (1951), Wiswell (1951) and Irons and Boyd (1952). These authors had claimed to have succeeded in controlling tumour growth in mice by injecting crude arginase solutions. However, in the meantime arginase preparations of higher purity have been made available (Bach and Killip, 1958 and 1961 ; Bach, Hawkins and Swaine, 1963). Such highly purified preparations when used in the present work for injections into rats bearing Walker carcinomas, resulted in a considerable retardation of tumour growth.

* Present address : School of Veterinary Science, University of Bristol, Langford, Bristol.

### MATERIAL AND METHODS

*Arginase preparations.*—The enzyme used in these experiments was prepared from ox liver according to a method similar to or identical with that of Bach, Hawkins and Swaine (1963); the preparations were taken to the "pre-crystallisation stage" of this method. The unit of arginase activity was defined as that amount of enzyme which liberates 1 $\mu$l urea—$CO_2$ (1/224 $\mu$ mole) from a 0·05 M L-arginine hydrochloride solution in 10 minutes at 30° and at pH 9·0. The specific activity of the enzyme solution was defined as activity units/mg. protein-nitrogen; the protein-nitrogen was determined spectrophotometrically (Bach and Killip, 1958).

*Animals.*—200–250 g. rats were used. They were albinos of the Chester Beatty strain and were fed throughout on standard laboratory diet.

*Implantation and measurement of tumours.*—The Walker 256 carcinoma was used throughout. Solid fragments of tumour were removed under sterile conditions from tumour-bearing rats and implanted into other rats by means of trochar and cannula under general anaesthetic. After 5–8 days the new tumour had reached a palpable size and was large enough for the experiments to commence. In each experiment 8–10 animals were assigned to both the control and experimental groups. The tumours were measured in cm. in two dimensions with calipers and the area ($cm.^2$) so obtained, constituted the tumour area.

*Injections.*—All injections (of usually 0·5 ml.) were made intra-peritoneally at daily, or at 2 day intervals.

*Enzyme dose.*—This varied considerably in the different experiments, ranging from 1,000 to 137,000 enzyme units in extreme cases.

*Statistical analysis.*—The significance of the difference between the changes in tumour size of the control and experimental animals was calculated for each experiment using the Student "$t$" test, and was expressed as probability "$p$".

The relative significance of the occurrence of an accelerating or retarding effect on tumour growth was assessed by a chi-squared test. Chi-square $= 2(-\ln p)$. Tables of chi-square were entered for the appropriate degrees of freedom, in this case, twice the number of experiments under consideration.

### RESULTS

The object of this study was to investigate the effect of injections of arginase solutions into tumour-bearing rats on the growth of their tumours.

*Compilation of the results in Table I.*—The results listed in Table I involved 18 experiments with a total of 360 rats, about half the number of which were injected intraperitoneally with highly purified arginase solutions, while the other half were injected with saline. The tumour areas of the controls and of the enzyme-injected rats were measured at intervals of 1, 2, 3 or 4 days after the first injection. In some of the 18 experiments the tumours were measured on each of the 4 days, in others only on some of these days. From the differences between the tumour areas of those measured on the day of the first injection and those measured on the 1st, 2nd, 3rd or 4th day after the injection, the changes in tumour areas were obtained both for the controls and the enzyme injected rats. These changes were averaged for each experiment (involving about 20 rats) and the mean changes so obtained are listed in Table I for each of the 4 days of observation. In this way, each of the pairs of values in Table I represents the mean area changes

TABLE I.—*The Effect of Arginase on Tumour Growth*

Mean changes in tumour area ($cm.^2$) ± S.E.M.

| Number of experiment | 1st Day after initial injection of: Saline | Arginase | p | 2nd Day after initial injection of: Saline | Arginase | p |
|---|---|---|---|---|---|---|
| 28 | 0·73±0·5 | −0·54±0·5 | 0·1 | 1·70±0·3 | 0·40±0·3 | 0·01 |
| 8a | 2·25±0·3 | 1·83±0·7 | >0·1 | 4·66±0·3 | 3·94±0·9 | >0·1 |
| 8b | 3·66±0·6 | 2·06±0·4 | >0·1 | 6·11±0·7 | 6·18±0·1 | >0·1 |
| 4 | 3·38±1·4 | 1·32±0·4 | >0·1 | 4·43±0·5 | 2·38±0·2 | 0·01 |
| 18 | 1·43±0·3 | 1·39±0·5 | >0·1 | 2·21±0·3 | 3·43±1·3 | >0·1 |
| 16 | 1·31±0·3 | 1·63±0·1 | >0·1 | 3·39±0·5 | 3·38±0·7 | >0·1 |
| 24 | 3·96±0·5 | 4·05±0·5 | >0·1 | 6·83±0·8 | 7·18±0·6 | >0·1 |
| 23 | 2·30±0·2 | 2·29±0·5 | >0·1 | 5·58±0·6 | 4·61±0·6 | >0·1 |
| 25 | 1·46±0·5 | 0·04±0·7 | >0·1 | 4·57±0·4 | 3·81±0·8 | >0·1 |
| 17 | 1·01±0·2 | 1·12±0·4 | >0·1 | 1·01±0·2 | 2·76±0·5 | 0·0002 |
| 19 | — | — | — | 1·38±0·5 | 0·86±0·2 | >0·1 |
| 12 | — | — | — | 3·30±0·81 | 0·12±0·4 | 0·0002 |
| 9 | — | — | — | 7·39±0·4 | 5·48±0·7 | 0·02 |
| 6 | — | — | — | 4·22±1·2 | 1·38±0·5 | 0·06 |
| 18a | 1·43±0·3 | 0·91±0·1 | 0·1 | — | — | — |
| | 2·17±0·3 (95 rats) | 1·55±0·4 (90 rats) | | 4·33±0·5 (133 rats) | 3·39±0·6 (127 rats) | |

| Number of experiment | 3rd Day after initial injection of: Saline | Arginase | p | 4th Day after initial injection of: Saline | Arginase | p |
|---|---|---|---|---|---|---|
| 28 | 3·77±1·2 | 1·40±0·5 | 0·06 | 3·44±0·7 | 1·40±0·5 | 0·02 |
| 8a | 6·91±0·8 | 4·72±0·9 | 0·09 | 10·64±1·3 | 5·52±1·3 | 0·01 |
| 8b | 7·65±1·0 | 7·26±1·0 | >0·1 | 8·71±1·2 | 10·30±1·4 | >0·1 |
| 4 | 7·14±0·9 | 4·40±0·7 | 0·03 | 8·83±1·2 | 5·90±1·1 | 0·1 |
| 18 | 3·43±0·3 | 4·54±1·3 | >0·1 | 5·13±0·4 | 6·33±1·8 | >0·1 |
| 16 | 5·78±0·7 | 5·43±0·3 | >0·1 | 7·75±0·8 | 7·35±0·3 | >0·1 |
| 24 | 8·58±1·6 | 9·28±1·1 | >0·1 | — | — | — |
| 23 | 7·85±0·9 | 7·50±1·2 | >0·1 | — | — | — |
| 25 | 7·23±0·5 | 6·58±0·7 | >0·1 | — | — | — |
| 19 | — | — | — | 2·58±0·9 | 2·12±0·5 | >0·1 |
| 12 | — | — | — | 6·88±0·7 | 4·30±0·9 | 0·07 |
| 9 | — | — | — | 11·85±0·5 | 9·93±0·9 | >0·1 |
| 6 | — | — | — | 7·44±1·4 | 4·52±0·8 | 0·1 |
| 15 | 4·45±0·1 | 2·17±0·6 | 0·003 | — | — | — |
| 11 | 5·61±0·6 | 1·53±0·6 | 0·0004 | — | — | — |
| 7 | 4·70±0·3 | 5·59±0·8 | >0·1 | — | — | — |
| | 6·11±0·5 (119 rats) | 4·84±0·7 (117 rats) | | 7·88±0·9 (92 rats) | 6·34±0·9 (103 rats) | |

The pairs of values constitute the mean changes in tumour area after 1–4 days of tumour growth as observed with the approximate 20 rats involved in each experiment, half of which had received daily injections with arginase solution. The probability p was calculated according to the Student *t*-test.

of the tumours in one particular experiment and at one particular period of tumour growth. Since, as mentioned above, the same group of rats could not always be used for all 4 days of observation, only 10–14 pairs of area changes (instead of 18), together with their S.E.M. values, will be found in each of the 4 sections of the table. The significance of the differences in the growth rates of the tumours between the two categories of treatment is expressed by the p value for each experiment and for each day of observation.

A typical experiment (Exp. No. 9) is presented in Table II which shows the mean tumour areas in the initial stage as well as 2 and 4 days after the first arginase injection.

TABLE II.—*Mean Tumour Areas of Saline Treated and Arginase treated Rats (Summary of a typical experiment, No. 9)*

| | Saline treated | Arginase treated | p |
|---|---|---|---|
| Number of animals | 17 | 18 | |
| Initial area (cm.$^2$) | $4 \cdot 56 \pm 0 \cdot 26$ | $4 \cdot 96 \pm 0 \cdot 23$ | — |
| 2 days treatment | $11 \cdot 99 \pm 0 \cdot 47$ | $10 \cdot 53 \pm 0 \cdot 86$ | — |
| 4 days treatment | $16 \cdot 48 \pm 0 \cdot 57$ | $14 \cdot 91 \pm 0 \cdot 84$ | — |
| *Differences* | | | |
| 0–2 days | $7 \cdot 42 \pm 0 \cdot 45$ | $5 \cdot 57 \pm 0 \cdot 71$ | 0·04 |
| 0–4 days | $11 \cdot 90 \pm 0 \cdot 52$ | $9 \cdot 95 \pm 0 \cdot 84$ | 0·06 |

*Interpretation of Table I.*—An examination of the results in Table I shows that the average increase in tumour area in *all* experiments carried out on each of the four days of observation, (i.e. the average growth rate of the tumours during a particular period), was markedly lower with the arginase-treated animals than with their controls. On the other hand, an examination of the *individual* experiments recorded in the four sections of Table I reveals that of the total of 47 mean changes listed, the injection of arginase apparently caused growth retardation only in 32 cases, while a growth acceleration was found in 10 experiments; in the remaining 5 experiments, where the difference in the mean change of tumour area between saline and enzyme-treated animals was lower than 0·9 cm.$^2$, the enzyme was considered to have no effect.

*Statistical significance of the results in Table I.*—Out of the 32 "effective" experiments, statistical significance can only be claimed for 11 for which, except for two values of 0·06, the probabilities were all 0·03 or even considerably lower.

*Table III.*—By applying the chi-squared test for the determination of the *comparative* significance of the retarding and the accelerating effects of arginase on tumour growth, it was found that for the 2nd, 3rd and 4th day of observation, the retardation of tumour growth by arginase was highly significant (Table III). In contrast to this, the results which showed an apparent accelerating effect of the enzyme were not statistically significant.

TABLE III.—*The Comparative Significance of the Accelerating and Retarding Effects of Arginase on Tumour Growth*

(Chi-squared Test)

| Period after first injection | Experiments showing: | Number of experiments | Number of rats | Probabilities P |
|---|---|---|---|---|
| Day 1 | Growth retardation | 8 | 110 | 0·90 |
| | Growth acceleration | 3 | 75 | 0·97 |
| Day 2 | Growth retardation | 10 | 193 | 0·002 |
| | Growth acceleration | 4 | 67 | 0·15 |
| Day 3 | Growth retardation | 9 | 172 | 0·0001 |
| | Growth acceleration | 3 | 64 | 0·54 |
| Day 4 | Growth retardation | 8 | 168 | 0·01 |
| | Growth acceleration | 2 | 27 | 0·43 |

Chi-squared was calculated from $\Sigma 2(-\ln p)$. P was found by entering tables of chi-squared for the appropriate degrees of freedom (twice the number of experiments). The values for p in experiments showing retardation or acceleration of tumour growth were taken from Table I.

*Enzyme concentration and growth-retarding effect* (Fig. 1).—Depending on the particular enzyme preparation, the enzyme concentrations in the solutions used for the injections varied substantially in the individual experiments. With this in mind, the results listed in Table I were examined for a possible influence of the enzyme concentration on the growth-retarding effect. For the purpose, the enzyme effects observed after 1, 2, 3 and 4 days were grouped together and the mean enzyme effect per day on tumour growth, i.e. the average daily arginase

FIG. 1.—The effect of the concentration of injected arginase solutions on the retardation of tumour growth.

effect, was calculated for each experiment ; experiments in which measurements had been taken on one single day only, were not included. When the enzyme effects of 14 out of the total of 18 experiments were plotted against the enzyme concentrations used in these experiments, a striking correlation was found between the two variables, in that the lower enzyme concentrations were more effective than the higher ones. The correlation coefficient was calculated and the best fitting line was drawn (Fig. 1) (Moroney, 1956).

*Specific activity of the enzyme and growth-retarding effect.*—When the specific activities (" purity ") of the enzyme solutions used for the injections were plotted against the growth-retarding effects, no correlation was found. On the contrary, the values were distributed at random.

### DISCUSSION

The results presented here, together with their statistical evaluation, indicated that daily intra-peritoneal injections of suitable concentrations of arginase were accompanied in the majority of cases by a noticeable retardation of tumour growth. The retardation was maintained at least for 4 days, though it could only be observed in 32 out of a total of 47 experiments, each involving about 20 tumour-bearing rats. One of the reasons for the failure of arginase to exert a retarding effect on tumour growth in the remaining 15 experiments may possibly be the high enzyme concentrations used in many of these cases, as will be discussed below. Thus the average retarding effect for *all* experiments was 20 %–30 %, depending on the period of tumour growth within the 4 day observation period. However, the retardation of tumour growth in the 32 " effective " experiments was 31 %–77 %, with the greater effects observed in the earlier periods of tumour growth. The retarding effect of the enzyme injections on the growth rate of the tumours could be expected to lead eventually to tumour regressions. However, the incidence of regression was found to be only insignificantly higher in the arginase-treated animals (26/177) than in the controls (20/183).

Though the statistical significance of the differences between the mean changes in tumour size of the control and the experimental animals in the " effective cases " was limited to only 11 experiments, the very high comparative significance of the retarding effects of the enzyme (chi-squared test), on the 2nd, 3rd and 4th day of tumour growth, as seen from Table III, can be considered encouraging. The failure to obtain more significant results in the individual experiments of Table I is understandable in view of the substantial biological variations ; these find expression in the very considerable differences in the sizes of the initial tumours of the 360 rats which varied in area from 0·4 $cm.^2$ to 13·5 $cm.^2$. The possibility of a connection between the initial tumour sizes and the chance of obtaining an arginase effect on the rate of tumour growth was explored. However, a statistical investigation revealed no such correlation.

*Effect of enzyme concentration.*—In contrast to the appreciable retardation of tumour growth observed after intraperitoneal injection of purified arginase solutions in comparatively low concentrations, the lack of efficacy of high enzyme concentrations has yet to be explained. It could be argued that this may be due to difficulties concerning the absorption of concentrated solutions of high molecular matter from the peritoneum. However, it appears that the absorption of such solutions and of particulate matter in the unanaesthetised animal occurs principally in the subphrenic region of the peritoneum where the passage for such matters is relatively free (Yoffey and Courtice, 1956). It is true though that the work of Allen and Raybuck (1960) indicates that the diaphragmatic lymphatic plexus may not play the only part in the absorption of peritoneal protein. Whether a further, as yet unknown, mechanism of peritoneal absorption would be less efficient in disposing of protein at higher concentrations than at lower ones, is a matter of speculation.

*Effect of impurities.*—The effectiveness of preparations with low enzyme concentrations containing a large proportion of non-enzymic material, could be interpreted as the result of an inhibiting action on tumour growth of certain impurities in the preparations. However, the fact that no correlation was found to exist between the specific activity (i.e. the purity) of the enzyme solutions used and the enzyme effect, precludes this possibility.

*Transport of the enzyme to and beyond the vascular system.*—There is no doubt that the intraperitoneally injected enzyme reaches the vascular system. Hawkins (1963) found that after a single injection of 80,000 units of arginase into rats, a considerable rise in plasma arginase levels was obtained after a period of 1–2 hours.

This activity disappeared after another 4–5 hours. In other experiments bovine xanthine oxidase of a molecular weight of 300,000, injected intraperitoneally into mice bearing spontaneous mammary carcinomas, was judged to have reached the liver and tumour tissue, since the levels of this enzyme were raised in both tissues proportionally to the quantity injected (Haddow, de Lamirande, Bergel, Bray and Gilbert, 1958).

Whether the large arginase molecules (molecular weight 138,000, Greenberg, 1954) would actually reach the cell cytoplasm of any tissue, is another question. Entrance into the cell membrane of large molecules is possible and pinocytosis is one mechanism by which this could occur. Ghose, Nairn and Fothergill (1962) injected rabbit serum proteins, labelled with a fluorescent dye, and detected the proteins in the cytoplasm of malignant tissue and of Kupffer cells, but not in the nuclei, although Watson (1959) had shown the existence of large pores in the nuclear membrane. However, exogenous DNA was found by Bensch and King (1961) to be incorporated not only into the cytoplasm but also into the nuclei of cells.

Even if administered enzyme were transported no further than to the intercellular space, it could reduce the level of arginine within the cell by setting up an " arginine gradient " through enforced destruction of that quantity of arginine which is exchanged across the cell membrane. This could lead to a depletion of arginine in the cytoplasm and, possibly, in the nucleus. In this way the synthesis of the histones of nucleoproteins, rich in basic amino acids, would be impaired and the rapid growth of malignant cells possibly checked.

### SUMMARY

1. Intraperitoneal injections of highly purified arginase preparations into rats bearing Walker carcinomas over a period of 4 days caused a 31 %–77 % retardation of tumour growth in the great majority of cases, with the higher effects occurring in the earlier periods. In a small number of cases no such effect or even a growth acceleration was observed.

2. Owing to the considerable biological variations within the initial sizes of the tumours of the 360 rats used, the retarding effects were statistically significant only in a minority of cases. However, when the comparative significance of the retarding and accelerating effects of the enzyme on tumour growths were investigated, a high statistical significance was found for the retardation and no significance for the acceleration.

3. Lower enzyme concentrations apparently exerted a greater retarding effect than higher ones. A possible connection between this correlation and the mode of absorption of high molecular matter from the peritoneum is discussed.

4. Since no correlation was found between the purity of the enzyme preparation and its retarding effect on tumour growth, the possibility of impurities being responsible for the retarding effect could be excluded.

5. It is suggested that the presence of arginase in the intercellular space may set up an " arginine gradient " which could lead to a depletion of tumour tissue of arginine with a resulting impairment of tissue growth.

One of us (S. J. B.) is greatly indebted to Professor J. S. Mitchell, F.R.S., Department of Radiotherapeutics in the University of Cambridge, for the continuous use of laboratory facilities and the technical assistance given by his Department over many years, and in particular, for the advice and encouragement received during this time. Our grateful thanks also go to Mr. E. A. King who conscientiously carried out all injections and tumour measurements for us. The statistical investigations were in the able hands of Miss Margaret Bartlett and Mr. M. W. Blee of W. D. and H. O. Wills, Bristol, who gave valuable advice on the interpretation of our results. The experimental data were collected over a number of years with the collaboration of several of our colleagues, in particular, of Dr. J. D. Killip, now on the staff of the Biochemical Journal, also of Professor C. G. Schmidt, University of Münster, Germany, and of Mr. R. A. Hawkins, M.Sc., Twyford Laboratories, London N.W.10. The authors gratefully acknowledge the financial aid given to them by the British Cancer Campaign for Research during the whole of the experimental period.

### REFERENCES

ALLEN, L. AND RAYBUCK, R. B.—(1960) *Anat. Rec.*, **137**, 25.
BACH, S. J., HAWKINS, R. A. AND SWAINE, D.—(1963) *Biochem. J.*, **89**, 263.
*Idem* AND KILLIP, J. D.—(1958) *Biochim. biophys. Acta*, **29**, 273.—(1961) *Ibid.*, **47**, 334.
*Idem* AND LASNITSKI, I.—(1947) *Enzymologia*, **12**, 198.
*Idem* AND MAW, G. A.—(1953) *Biochim. biophys. Acta*, **11**, 69.
*Idem* AND SIMON-REUSS, I.—(1953) *Ibid.*, **11**, 396.
BENSCH, K. G. AND KING, W. K.—(1961) *Science*, **133**, 381.
BERGEL, F.—(1961) "Chemistry of Enzymes in Cancer". Springfield, Illinois, U.S.A. (Charles C. Thomas) p. 58.
GHOSE, T., NAIRN, R. C. AND FOTHERGILL, J. E.—(1962) *Nature, Lond.*, **196**, 108.
GREENBERG, D. M.—(1954) *Arch. Biochem. Biophys.*, **62**, 454.
*Idem* AND SASSENRATH, E. N.—(1953) *Cancer Res.*, **13**, 709.
HADDOW, A., DE LAMIRANDE, G., BERGEL, F., BRAY, F. AND GILBERT, R. C.—(1958) *Nature, Lond.*, **182**, 1144.
HAWKINS, R. A.—(1963) M.Sc. Thesis, Bristol University.
IRONS, W. G. AND BOYD, E. F.—(1952) *Ariz. Med.*, **9**, 39.
MORONEY, M. J.—(1956) "Facts from Figures". 3rd Edition. (Harmondsworth, England Penguin Books), p. 284.
VRAT, V.—(1951) *Permanente Fdn. med. Bull.*, **9**, 56.
WATSON, M. L.—(1959) *J. biophys, biochem. Cytol.*, **6**, 147.
WISWELL, O. B.—(1951) *Proc. Soc. exp. Biol. N.Y.*, **76**, 588.
YOFFEY, J. M. AND COURTICE, F. L.—(1956) 'Lymphatics, Lymphs and Lymphoid Tissue'. London (Edward Arnold).